# Emergence of oriental theileriosis in cattle and its transmission through *Rhipicephalus (Boophilus) microplus* in Assam, India

**DOI:** 10.14202/vetworld.2015.1099-1104

**Published:** 2015-09-22

**Authors:** Parikshit Kakati, Prabhat Chandra Sarmah, Debdatta Ray, Kanta Bhattacharjee, Rajeev Kumar Sharma, Luit Moni Barkalita, Dipak Kumar Sarma, Bhaben Chandra Baishya, Pranjal Borah, Bobitha Stanley

**Affiliations:** 1 Department of Parasitology, College of Veterinary Science, Assam Agricultural University, Guwahati, Assam, India; 2 Division of Parasitology, Indian Veterinary Research Institute, Izatnagar, Uttar Pradesh, India; 3 Department of Microbiology, College of Veterinary Science, Assam Agricultural University, Assam, India; 4 Department of Animal Biotechnology, College of Veterinary Science, Assam Agricultural University, Assam, India; 5 Teaching Veterinary Clinical Complex, College of Veterinary Science, Assam Agricultural University, Assam, India; 6 Goat Research Station, Burnihat, Assam Agricultural University, Assam, India

**Keywords:** *Anaplasma marginale*, Assam (India), *Babesia bigemina*, Ikeda type, *Rhipicephalus (Boophilus) microplus*, *Theileria orientalis*

## Abstract

**Aim:**

The aim of the present study was to investigate the presence of *Theileria* in blood samples of crossbred and indigenous adult cows raised under unorganized small scale farming system in a *Babesia* and *Anaplasma* endemic geographical area from Assam, India and to see its transmission through *Rhipicephalus* (*Boophilus*) *microplus* ticks.

**Materials and Methods:**

For the present study, 57 clinical cases of cattle suspected to be of hemoparasitic infections were taken into consideration. The parasites were identified based on morphology in giemsa stained blood smear followed by polymerase chain reaction (PCR). Sera samples were tested for *T. annulata* antibodies in plate and Dot-ELISA. PCR was also conducted in eggs of *Rhipicephalus (Boophilus) microplus* tick collected from a *Theileria orientalis* positive animal.

**Results:**

PCR amplified 1124, 776, and 160 bp DNA fragments of *B. bigemina* (64.91%), *T. orientalis* (21.05%) and *A. marginale* (14.03%), respectively. This assay further conducted in 12 *T. orientalis* positive blood samples with primers of Buffeli, Chitose, and Ikeda variants of *T. orientalis* showed 3 samples positive to Ikeda type and none for Buffeli and Chitose. *Babesia bovis* and *Theileria annulata* specific primers also did not amplify any fragment during the PCR assay of the blood samples. Further, all sera samples tested negative to *T. annulata* antibodies in Plate and Dot-ELISA. PCR conducted in eggs of *R (B).microplus* tick collected from a *T. orientalis* positive animal revealed presence of the parasite DNA. Gradual improvement in physical condition leading to complete recovery in 10 out of 12 *T. orientalis* infected clinical cases treated with buparvaquone(at 2.5mg/kg.b.wt I/M) was the feedback obtained from field veterinarians and the cattle owners.

**Conclusion:**

The present investigation represents the first report of occurrence of *T. orientalis* in cattle of Assam with involvement of pathogenic Ikeda strain in clinical outbreaks and its possible natural transmission by *R (B). microplus* through the transovarian mode.

## Introduction

*Theileria orientalis*, a hemoprotozoan parasite of cattle and the causative agent of oriental theileriosis in the Asian countries in former times has now been reported from different countries in the tropics and sub-tropics. It is believed to be transmitted transtadially through different species of *Haemaphysalis* ticks [1,2]. The parasite was previously considered non-pathogenic since it caused mild anemia in cattle of endemic areas [2]. However, in the recent past the parasite has emerged pathogenic as evidenced by a number of clinical outbreaks characterized by fever, anemia, jaundice and abortion and even mortality recorded from several countries [3,4]. In India also, the parasite was considered non-pathogenic [5] for which not much attention was paid until recent record of fatal disease due to *T. orientalis* in crossbred adult bovines infested with *Haemaphysalis bispinosa* in Southern India [6].

Assam, situated in the Northeast region of India is known to have problems of tick infestation predominantly with *Rhipicephalus (Boophilus) microplus* [7] and prevalence of *Babesia* and *Anaplasma* in cattle [8]. There was essentially no local knowledge on the prevalence of *Theileria* species in the state although *Theileria annulata* causing bovine tropical theileriosis had been reported from neighboring states of Tripura and West Bengal [9,10]. In the recent past, veterinary practitioners from Assam have been experiencing a poor response to specific treatment in several field cases of *Babesia* like infections in cross-bred cattle.

A systematic study was therefore conducted using clinical, blood smear, serological, and molecular analysis in clinical outbreaks of hemoparasitic infection to address the issue, and the results are presented through this communication.

## Materials and Methods

### Ethical approval

The experiments comply with the guidelines laid down by the Institutional Ethical Committee and in accordance with the country law.

### Study area

The study was conducted in the Southeast peri-urban area of Guwahati under Kamrup (Metropolitan) District of Assam, India. Jersey and Holstein Friesian crossbred cattle are being raised under a stall-fed unorganized farming condition in this area from which major part of milk is supplied to the city.

### Study population

During May, 2012 to April, 2013, field veterinarians identified 57 clinical cases suspected to be of hemoparasitic infection on the basis of clinical symptoms such as pyrexia, anorexia, pale mucous membrane, and in severe cases jaundice. The clinical cases were attended individually for physical examination including the search for ticks on the body parts. Ticks were collected in glass vials for their identification [11-13].

### Hematological analysis

The blood samples (n=57) were collected in tubes with or without EDTA for parasitological, molecular, and serological studies. Giemsa stained smears prepared from superficial lymph node aspirations, and fresh blood samples were examined under a microscope for detection of parasites and in positive cases, parasitemia was estimated.

### Molecular study

Genomic DNA was extracted from whole blood using DNeasy Blood and Tissue Kit (Quiagen; Catalogue No. 69504) as per manufacturer’s protocol. PCR assay was carried out in a Techne-500 thermal cycler (Bibby Scientific) using established primers for *Theileria annulata* and *T. orientalis* with its variants *viz*. Chitose, Buffeli, and Ikeda using respective protocols for preparation of reaction mixture and thermocycling conditions [2,14] (Table-1). Additionally, established primers of *Babesia bigemina*, *Babesia bovis*, and *Anaplasma marginale* were also used to confirm their identity in clinical cases vis a vis differential diagnosis through molecular evaluation [15-17]. PCR products were confirmed by electrophoresis in 1.5% agarose gel prestained with Ethidium Bromide and subsequent visualization done in gel documentation system (DNR Mini Lumi, Applied Bioimaging).

**Table-1:** Standard primers used for identification of *T. annulata* and *T. orientalis* (including type chitose, buffeli, and ikeda) along with their primer sequences, amplification targets, cycling conditions, and product size.

Parasite	Primer sequence pair	Amplification target	Cycling conditions	Product size
*T. annulata*	TaF1:5’-GTA ACC TTT AAA AAC GT-3’ TaR1:5’-GTT ACG AAC ATG GGT TT-3’	30 kDa major *T.annulata* merozoite surface antigen gene	94°C, 1 min 55°C, 1 min 72°C, 1 min 30 cycles	721 bp
*T. orientalis (Entire* *T. orientalis group)*	Tor: F1:5’-CTT TGC CTA GGA TAC TTC CT-3’ Tor: R1:5’-ACG GCA AGT GGT GAG AAC T-3’	Gene encoding a polymorphic MPSP	95°C,15 sec 57°C,30 sec 72°C, 1 min 30 cycles	776 bp
*T. orientalis* *(Type-Chitose)*	Tor^C^: F1:5’-GCG GAT CCT CAT CGT CTC TGC AAC T-3’	SSUrRNA gene		831 bp
*T.orientalis* *(Type-Buffeli)*	Tor^B^: F1:5’-GCG GAT CCG CTC TGC AAC CGC AGA G-3’			825 bp
*T.orientalis (Type-Ikeda)*	Tor^I^: F1:5’-AAG GAT CCG TCT CTG CTA CCG CCG C-3’			826 bp

T. orientalis=Theileria orientalis, T. annulata=Theileria annulata, MPSP=Merozoite/piroplasms surface protein

### Serological study

Sera samples of all clinical cases were subjected to Plate and Dot-ELISA [18] at the Division of Parasitology, Indian Veterinary Research Institute, Izatnagar to screen for the presence of *T. annulata* antibodies.

A fully engorged adult *R (B). microplus* tick collected from a clinical case positive to *T. orientalis* was placed for oviposition inside a dessicator at room temperature. The DNA extracted from the egg masses in triplicate were employed in PCR analysis using primers and protocols for *T. orientalis*.

Twelve cattle that were test positive for *T. orientalis* based on molecular diagnosis received treatment with a single injection of buparvaquone at 2.5 mg/kg body weight intramuscularly. Clinical cases of babesiosis and anaplasmosis were treated with diminazene aceturate at 3.5 mg/kg body weight intramuscularly for one occasion each and oxytetracycline at 5.0 mg/kg body weight 12 hourly intravenous for 5 days respectively. Feedback on post-treatment recovery was obtained from the field veterinarians and the cattle owners for confirmation and to exclude non-parasitic causes of hemolytic anemia.

## Results

**Figure-1:**
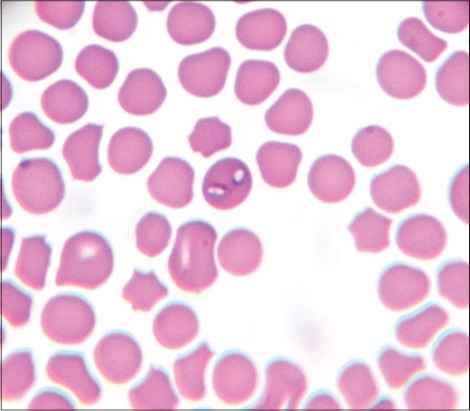
Giemsa stained blood smear showing paired pear-shaped red blood cell form of *Theileria orientalis* (×1000).

**Figure-2:**
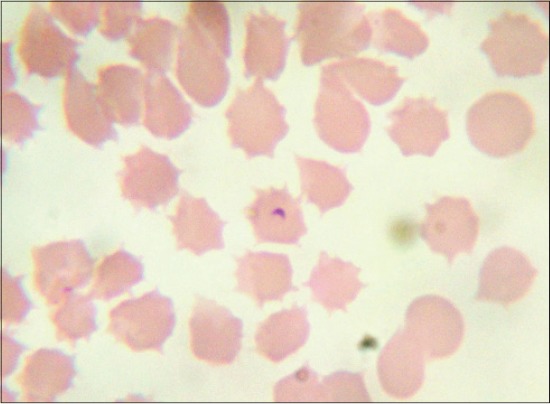
Giemsa stained blood smear showing crescent shaped the intraerythrocytic form of *Theileria orientalis* (×1000).

**Figure-3:**
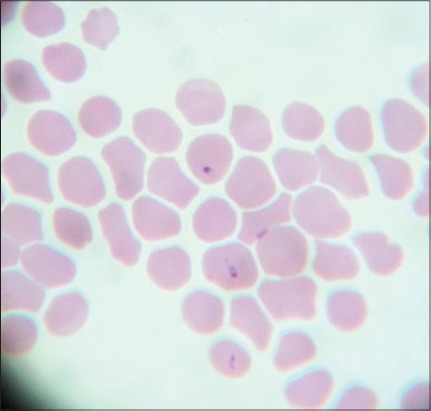
Giemsa stained blood smear showing the intraerythrocytic rod of *Theileria orientalis* (×1000).

**Figure-4:**
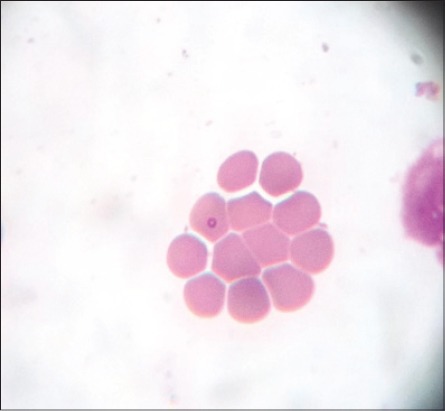
Giemsa stained blood smear showing dot shaped the intraerythrocytic form of *Theileria orientalis* (×1000).

**Figure-5:**
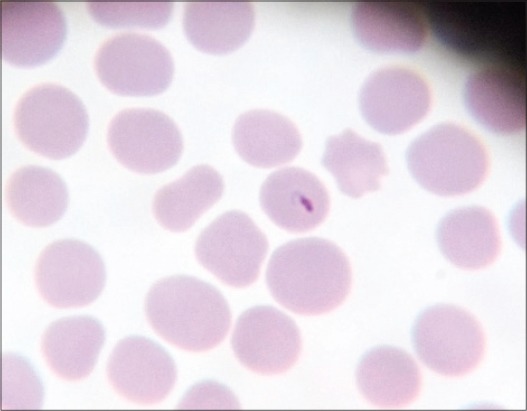
Giemsa stained blood smear showing comma shaped the intraerythrocytic form of *Theileria orientalis* (×1000).

**Figure-6:**
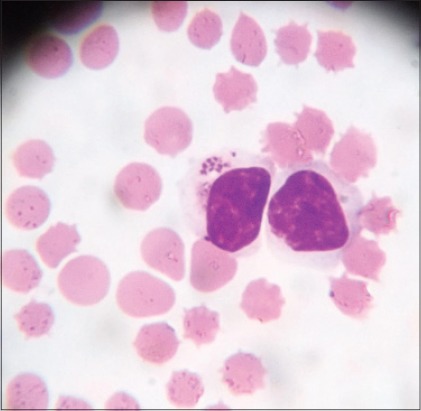
Giemsa stained blood smear showing intralymphocytic schizont of *Theileria orientalis* (×1000).

Microscopic examination of blood smears showed overall 89.47% positivity to hemoparasites. These included a large form of *Babesia* in 33 cases, *Anaplasma* in 8 cases and *Theileria* like organisms in 12 cases. *Theileria* like organisms inside the erythrocytes were small pear shaped (Figure-1), crescent (Figure-2), rod (Figure-3), dot (Figure-4), and comma-shaped (Figure-5) with trailing cytoplasm. The erythrocytic parasitemia in all positive cases was <1%. In a few cases, lymphocytes showed the presence of intracytoplasmic schizonts (Figure-6).

**Figure-7:**
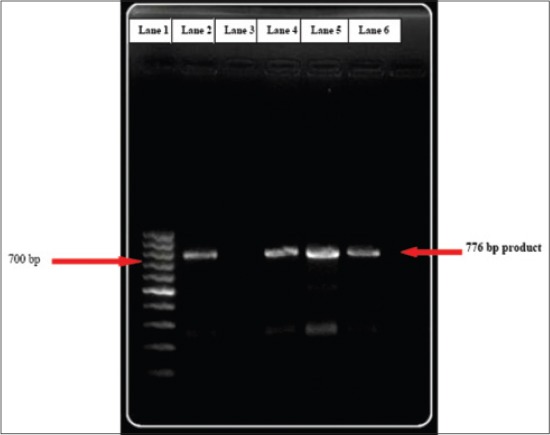
776 bp fragments of *T. orientalis* DNA in 1.5% agarose gel. (Lane 1: 100 bp DNA Marker, Lane 2: Positive control, Lane 3: Negative control, Lane 4, 5, 6: Test samples).

**Figure-8:**
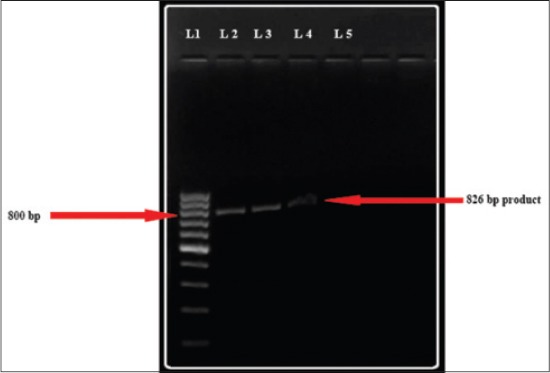
826 bp fragments of *Theileria orientalis* (type ikeda) DNA in 1.5% agarose gel. (Lane 1: 100 bp DNA Marker, Lane 2, 3, 4: Test Samples, Lane 5: Negative control).

Polymerase chain reaction amplified 1124, 160 bp and 776 bp DNA fragments of *B*. *bigemina* (n=33, 64.91%), *A. marginale* (n=8, 14.03%) and *T. orientalis* (n=12, 21.05%) (Figure-7) respectively. PCR analysis of *T. orientalis* positive DNA extracts using variant-specific primers, showed 3 animals positive to Ikeda variant with a product size of 826 bp (Figure-8). *T. annulata* and *B. bovis* specific primers did not amplify the desired fragments in PCR.

Plate-ELISA and Dot-ELISA tests performed in sera of all the clinical cases using *T. annulata* antigen were all negative for homologous antibodies.

**Figure-9:**
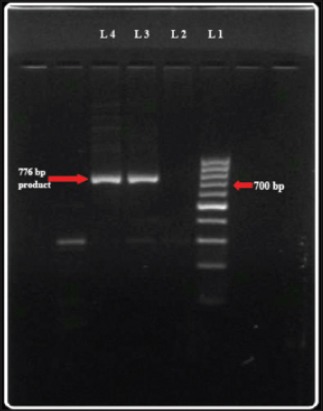
776 bp fragments of *Theileria orientalis* DNA in *Rhipicephalus* (*Boophilus*) *microplus* egg in 1.5% agarose gel. (Lane 1: 100 bp DNA Marker, Lane 2: Negative control, Lane 3: Positive control, Lane 4: Test sample).

Ticks present on the bodies of animals were all identified as *R (B). microplus*. PCR assay conducted on the DNA extracted from eggs of the tick also amplified *T. orientalis* specific primers (Figure-9).

Clinical symptoms observed in diseased cattle were fever (104-106°C) with varying degrees of pale mucous membrane, anorexia, depression, dehydration, weakness, and recumbency. Dark urine and icterus were other prominent clinical signs. Animals with *T. orientalis* infection in severe cases passed tarry colored dung.

Adoption of parasite-specific treatment showed gradual improvement in health with the disappearance of clinical symptoms within 48 h of treatment except mortality recorded in 2 animals with *T. orientalis* infection.

## Discussion

Assam is situated in the central part of the Northeast India (between 24°8′ and 28°2′ N latitude and 89°42′ and 96°E longitude). The state experiences moderately hot and very humid climate with a total annual rainfall of about 2818 mm favoring the propagation of vectors and many vector-borne diseases of man and animals. This state is already known to be endemic for *Babesia* and *Anaplasma* of cattle [8]. Investigation of the parasitic etiology of hemolytic anemia conducted in the present study revealed the presence of erythrocytic piroplasms of *Theileria* like organisms [19] in blood smears and rare detection of intracytoplasmic schizont within lymphocytes. Consistent with the previous report [8], *Babesia* and *Anaplasma* were also detected in blood smears of affected cattle. Light microscopy is widely used in the most of the laboratories to gather important diagnostic informations characteristic to a particular species of hemoparasite. However, it seems to be a difficult task in field cases of natural infection to differentiate small forms of *Babesia* (*B. bovis*), *Anaplasma* and closely related *Theileria* species which have some common morphological appearances. The recent adoption of molecular techniques has made a strong impact in the field of diagnostic parasitology in supporting surveillance, monitoring and disease management programs. Molecular studies conducted in blood samples of present study confirmed the presence of *T. orientalis*, *B. bigemina*, and *A. marginale* and at the same time ruled out association of *T. annulata* and *B*. *bovis* consistent with previous report made from Kerala [20]. First record of *Theileria*, the *T. orientalis* in cattle of Assam and its association with clinical outbreaks is on the line of experiences gathered by the field veterinarians who observed variable response to treatment with diminazene compounds in animals with clinical *Babesia* like infections. Similar reports on the prevalence of *T. orientalis*, its association with clinical outbreaks with fatal results among adult cattle have been reported recently from South India [20]. Present findings also conformed to the previous reports of *T. orientalis* made from other countries [2,3,21-24]. *T. orientalis* populations are known to consist of 8 genotypes of which involvement of Ikeda group in the production of the clinical disease have been proved by many workers [2,4,25]. Recent reports of Ikeda genotype of *T. orientalis* from clinical outbreaks in cattle of Australia having no history of cattle importation has also pointed out possible emergence of this variant from the existing Buffeli type through silent mutation [2] and possible existence of gene switching [26]. PCR analysis conducted further in *T. orientalis* positive blood samples in the present study using established primers of Ikeda, Buffeli, and Chitose variants showed the presence of only Ikeda genotype in 3 positive cases. This suggested the involvement of some other variants of *T. orientalis* thus warranting further confirmation and subsequent phylogenetic analysis in order to establish the different genotypes of parasite species prevalent in Assam.

Epidemiological studies conducted by several workers in different parts of India revealed a high prevalence of *T. annulata* [10,27]. However, PCR analysis in the present study using *T. annulata* specific primers failed to amplify parasite DNA in the blood samples. *T. annulata* antibody negative results obtained in the serological test conducted in sera of *T. orientalis* PCR positive cattle also confirmed the absence of *T. annulata* in Assam. This correlates with the previous report on the absence of *Hyalomma anatolicum*, the proven vector of *T. annulata* in cattle from Assam [7,28]. Similar situation on the absence of *Hyalomma* tick and *T. annulata* in Kerala was described [6] inspite of their prevalence record in neighboring states like Tamil Nadu and Karnataka.

PCR conducted in the DNA extracted from eggs laid by engorged *R (B).microplus* in this study revealed presence of *T. orientalis* DNA which suggested that the tick might be the vector of *T. orientalis* infection transmitting the parasite through transovarian mode. Further investigations on tick vector are required using large sample size to authenticate the present finding. Epidemiology of *T. orientalis* and its vector have not been adequately studied in India. However, *Haemaphysalis bispinosa* was recovered from *T. orientalis* infected cattle [6] similar to reports of *H. longicornis* and other species of *Haemaphysalis* as a vector with transtadial transmission [1,2]. The reports on experimental transmission of *Theileria mutans* [29] and *T. equi* [30] by *B. microplus* might justify the potential of this tick to transmit *T. orientalis* under natural conditions via transovarian route. An epidemiological situation similar to the present study was also reported from Vietnam [23] where the prevalence of *T. orientalis* was on record in cattle infested with only *B. microplus* ticks.

In addition to analysis of clinical findings in *T. orientalis* infected cases our aim was also to obtain feedback from the practitioners on the response of animals to administration of buparvaquone which was marketed in Assam (Northeast India) soon after a diagnosis of *T. orientalis* infection was made. Animals in the early stage of infection showing fever, depression and inappetence responded well to treatment with buparvaquone. Consistent with previous reports [31], response to buparvaquone, as observed in the present study, was another confirmatory diagnosis to rule out non-parasitic causes of anemia which might have prevailed in animals under natural conditions. However, similar treatment provided to two *Theileria* positive animals in a state of recumbency and showing tarry colored dung was non-responsive and turned fatal. Although no post-mortem could be conducted in field cases, treatment failure in these 2 animals suggested severe hemorrhagic condition in the gastrointestinal tract similar to earlier record [6] of punched out ulcers in the abomasum and hemorrhagic duodenitis in cattle with *T. orientalis* fatal infection.

## Conclusion

The findings of the study represent the first report on the occurrence of *T. orientalis* and involvement of pathogenic strain responsible for the clinical disease of emerging nature in Assam, India. Detection of *T. orientalis* DNA in the eggs of *R (B)microplus* is another important finding that justifies the significant role of this highly prevalent vector tick species in the epidemiology of *T. orientalis* along with *B. bigemina* and *A. marginale* in Assam. Future studies on phylogenetic analysis and experimental tick transmission of *T. orientalis* in cattle should provide additional information in understanding the complete epidemiology of the parasite in the Northeast region of India including Assam.

## Authors’ Contributions

This study was a part of PK’s MVSc dissertation under the advisory committee of PCS, KB and RKS. DKS, BCB, and PB provided samples from clinical cases and treatment feedback. DR and BS performed serological works at IVRI. LMB assisted in molecular works. PCS, KB, and PK drafted the manuscript with subsequent revision. All authors read and approved the final manuscript.
